# Flexible Stretchable Strain Sensor Based on LIG/PDMS for Real-Time Health Monitoring of Test Pilots

**DOI:** 10.3390/s25092884

**Published:** 2025-05-02

**Authors:** Shouqing Li, Zhanghui Wu, Hongyun Fan, Mian Zhong, Xiaoqing Xing, Yongzheng Wang, Huaxiao Yang, Qijian Liu, Deyin Zhang

**Affiliations:** 1 Civil Aviation Administration of China Academy, Civil Aviation Flight University of China, Deyang 618307, China; sqli@cafuc.edu.cn; 2 College of Aviation and Electronics and Electrical, Civil Aviation Flight University of China, Deyang 618307, China; wu3732596zh@163.com (Z.W.); 18731838513@163.com (H.F.); xingxiaoqing@cafuc.edu.cn (X.X.); morrisvip@cafuc.edu.cn (D.Z.); 3 Key Laboratory of Flight Techniques and Flight Safety, Civil Aviation Administration of China, Deyang 618307, China; 4 Civil Aviation Flight Test Institute, Civil Aviation Flight University of China, Deyang 618307, China; wangyongzheng@cafuc.edu.cn; 5 Mianyang Branch, Civil Aviation Flight University of China, Mianyang 621000, China; huaxiaoyang@cafuc.edu.cn; 6 College of Computer Science, Civil Aviation Flight University of China, Deyang 618307, China; qijian@cafuc.edu.cn

**Keywords:** flexible wearable sensor, laser-induced graphene, PDMS, real-time monitoring, stretchable strain sensor

## Abstract

In the rapidly advancing era of intelligent technology, flexible strain sensors are emerging as a key component in wearable electronics. Laser-induced graphene (LIG) stands out as a promising fabrication method due to its rapid processing, environmental sustainability, low cost, and superior physicochemical properties. However, the stretchability and conformability of LIG are often limited by the substrate material, hindering its application in scenarios requiring high deformation. To address this issue, we propose a high-performance flexible and stretchable strain sensor fabricated by generating graphene on a polyimide (PI) substrate using laser induction and subsequently transferred onto a polydimethylsiloxane (PDMS). The resultant sensor demonstrates an ultra-low detection limit (0.1%), a rapid response time (150 ms), a wide strain range (40%), and retains stable performance after 1000 stretching cycles. Notably, this sensor has been successfully applied to the real-time monitoring of civil aviation test pilots during flight for the first time, enabling the accurate detection of physiological signals such as pulse, hand movements, and blink frequency. This study introduces a unique and innovative solution for the real-time health monitoring of civil aviation test pilots, with significant implications for enhancing flight safety.

## 1. Introduction

Advances in composite materials and nanotechnology have increasingly facilitated the civilianization of health monitoring, thereby promoting the rapid development of wearable sensors [1,2,3]. Wearable electronic devices are capable of real-time and accurate monitoring of human health indicators by receiving various biological or physiological stimuli, and have garnered significant attention from scholars in recent years [4,5,6,7]. Among these, flexible strain sensors exhibit excellent stretchability, extensibility, and universality and have experienced rapid development [8,9]. The primary types of flexible strain sensors include piezoelectric [10], capacitive [11], and piezoresistive [12] sensors. Piezoresistive strain sensors quantify external strain through resistance changes. To facilitate their application in human health monitoring, researchers have investigated numerous sensing materials with superior properties and biocompatibility, such as hydrogels [13,14], MXene [15,16], graphene [17,18], and other materials. Among these materials, graphene is particularly noteworthy due to its exceptional optical [19], electrical [20], and mechanical [21] properties, as well as its two-dimensional flexible porous structure [22], making it highly significant for research in the field of flexible sensing.

Currently, the preparation methods for graphene include the mechanical exfoliation method [23], chemical vapor deposition (CVD) [24], liquid-phase exfoliation [25], and other techniques. Nevertheless, these methods are either economically burdensome, environmentally unfriendly, or result in low-quality graphene. For instance, the mechanical exfoliation method is capable of producing high-quality, defect-free graphene but faces challenges in scaling up for mass production [26]. The CVD method enables large-scale graphene synthesis but involves high costs, complex processes, significant environmental pollution, and inconsistent yields [27]. Laser-induced graphene (LIG), a groundbreaking technology that emerged in 2014 [28], generates graphene through in situ modification and customizable patterning to satisfy various application requirements. This innovation has facilitated the development of a series of LIG-based sensors [29]. LIG exhibits excellent electrical conductivity and can be transferred onto other substrates to enhance their tensile properties, making it highly promising for wearable electronic applications [30]. Precursor materials play a critical role in determining the morphology and properties of the generated graphene. Common precursors include polyimide (PI), polydimethylsiloxane (PDMS), and PET. Under laser irradiation, these materials undergo the cleavage of their chemical bonds, releasing nitrogen (N) and oxygen (O) elements in gaseous forms, while the remaining carbon (C) atoms rearrange to form LIG [31]. However, PI exhibits a maximum strain before fracture that is less than 5%, which is insufficient compared to the allowable strain of human skin, exceeding 13%. This limitation restricts the wearing comfort and applicability of sensors based on PI substrates [32]. PDMS, renowned for its biocompatibility and chemical stability, exhibits a typical strain range of 40–60% in practical applications. PDMS’s tensile properties significantly surpass the requirements of the human body. To enhance the stretchability of the sensors, scholars have investigated various research methods. For instance, Wang et al. [33] incorporated PI particles into PDMS for solidification followed by subsequent LIG generation, achieving a stretchability of up to 15%. Additionally, Tahir et al. and other researchers have applied LIG transferred onto PDMS for motion stretch detection [34,35].

The key innovations and contributions of this study can be summarized as follows:

1. By integrating laser direct writing technology and the transfer method, high-performance LIG was successfully fabricated on PI and subsequently transferred to a PDMS substrate, creating an LIG/PDMS flexible strain sensor with excellent tensile properties and sensitivity. This sensor demonstrates a maximum tensile strain of approximately 40% and a gauge factor of 20.7 within a 21% strain range.
2. The LIG/PDMS-based flexible strain sensor exhibits outstanding sensing characteristics, including superior electrical conductivity (48 Ω), a low detection limit (0.1%), rapid response and recovery times (150 ms/200 ms), and excellent durability after undergoing more than 1000 stretching cycles.
3. This LIG/PDMS-based flexible strain sensor was innovatively applied for the first time in the real-time monitoring of civil aviation test pilots, capturing physiological signals including blinking, pulse, finger bending, and wrist movement. This application provides a novel, efficient, and comfortable approach for real-time pilot monitoring.

## 2. Materials and Methods

### 2.1. Preparation of LIG Strain Sensor

**Figure 1:**
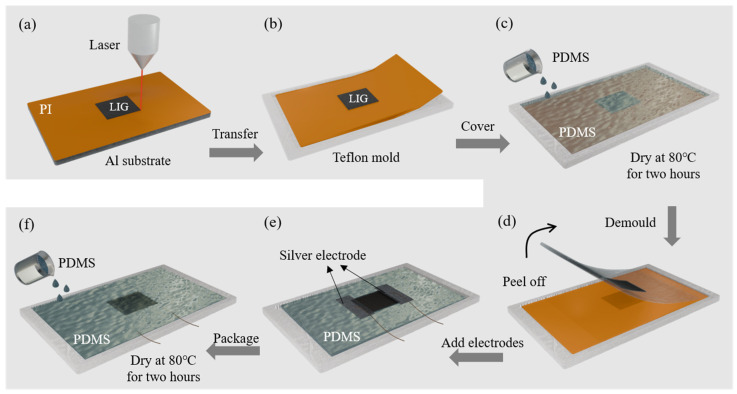
Preparation method of the LIG/PDMS flexible stretchable strain sensor: (**a**) laser direct writing to produce graphene; (**b**) trimming PI and placing it in a mold; (**c**) pouring PDMS curing; (**d**) stripping PDMS film with LIG layer from PI film; (**e**) adding electrodes; (**f**) repeating step c.

The preparation process of the LIG/PDMS-based flexible stretchable strain sensor is illustrated in Figure 1. Initially, a 125-μm-thick PI film (provided by Shenzhen Jihongda Plastic Products Co., Ltd., Shenzhen, China) was adhered to a metal substrate, as shown in Figure 1a. Subsequently, the PI film was subjected to line sweeping using a CO_2_ infrared laser scribing (Synrad P150, Novanta Corporation, Bedford, MA, USA). The laser parameters were set as follows: power of 2.3 W, scanning speed of 100 mm/s, and line width of 200 μm. This procedure generated striped patterns, ultimately forming 10 mm × 10 mm graphene blocks. The resulting graphene exhibited a porous structure and was primarily single-layered. Cube slots with dimensions of 50 mm × 30 mm × 500 μm were fabricated using a computer numerical control (CNC) lathe, with Teflon serving as the material for this step. The PI film with graphene was then cut to match the mold size and positioned inside it, as illustrated in Figure 1b, ensuring that the graphene stripes were aligned parallel to the 50 mm sides of the mold.

SYLGARD 184 silicone elastomer (DOW) was mixed with its crosslinker at a ratio of 10:1 and homogenized using a blender for 5 min to prepare a PDMS solution. The solution was subsequently degassed in a vacuum dryer for 2 h to eliminate bubbles introduced during stirring. The PDMS solution was then carefully poured into the mold depicted in Figure 1c, ensuring uniformity of the mold to maintain consistent sensor thickness. The mold was cured in a vacuum drying oven at 80 °C for 2 h. On the following day, after complete curing, the upper layer of PDMS was carefully peeled off, as shown in Figure 1d. Conductive silver paste was applied to both sides of the graphene, and electrical wires were connected (Figure 1e). Step 1c was then repeated to encapsulate the opposite side of the sensor with PDMS, thereby completing the full encapsulation (Figure 1f).

### 2.2. Materials Characterization

**Figure 2:**
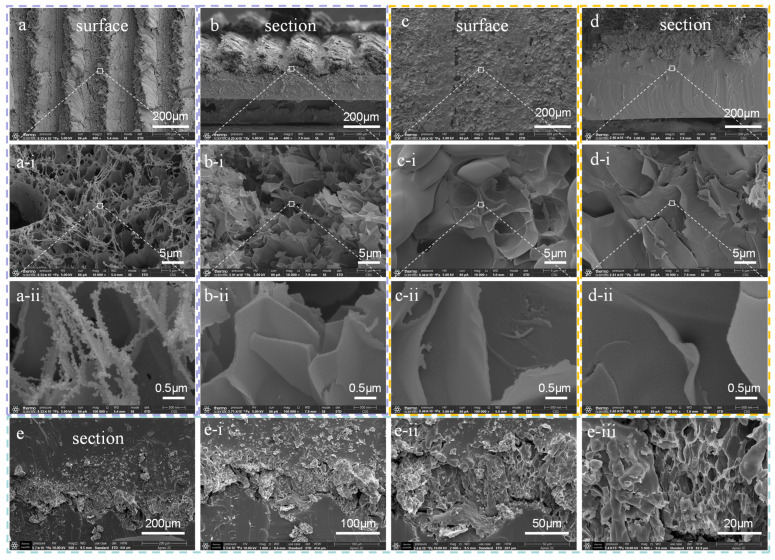
SEM images of the LIG/PDMS-based sensor fabrication process: (**a**,**b**) graphene generated by laser ablation on PI; (**c**–**e**) LIG after transfer to PDMS. (**a**-**i**), (**a**-**ii**) Show 40× and 400× magnified views of (**a**), respectively. Similarly, (**b**-**i**), (**b**-**ii**), (**c**-**i**), (**c**-**ii**), (**d**-**i**), (**d**-**ii**) correspond to the same magnification levels for (**b**–**d**). (**e**-**i**), (**e**-**ii**), (**e**-**iii**) Present 2×, 4×, and 10× magnified views of (**e**), respectively.

The surface topography of LIG on PI, the graphene transfer process, and the characteristics of the transferred sensor were systematically analyzed. Figure 2 shows that the scanning electron microscopy (SEM) images reveal multiple stages of the sensor fabrication process, allowing for detailed observation of both its surface morphology and cross-sectional structure. It is shown in Figure 2a,b that the SEM images depict LIG formed on the surface and cross-section of the PI substrate. Additionally, the SEM images confirm that graphene exhibits a triangular morphology above the PI substrate, with striped scanning lines and a three-dimensional porous structure. This unique structure is responsible for the high sensitivity of the LIG-based sensor. Figure 2c,d demonstrate that the SEM images, which highlight the graphene after being successfully transferred onto the PDMS substrate. It is evident that PDMS and graphene are well-combined without obvious gaps. The cross-sectional view of the sensor, fully encapsulated by PDMS, is shown in Figure 2e. At this stage, the sensor retains the three-dimensional porous structure of graphene, ensuring its sensitivity while significantly enhancing its stretchability compared to the original configuration.

**Figure 3:**
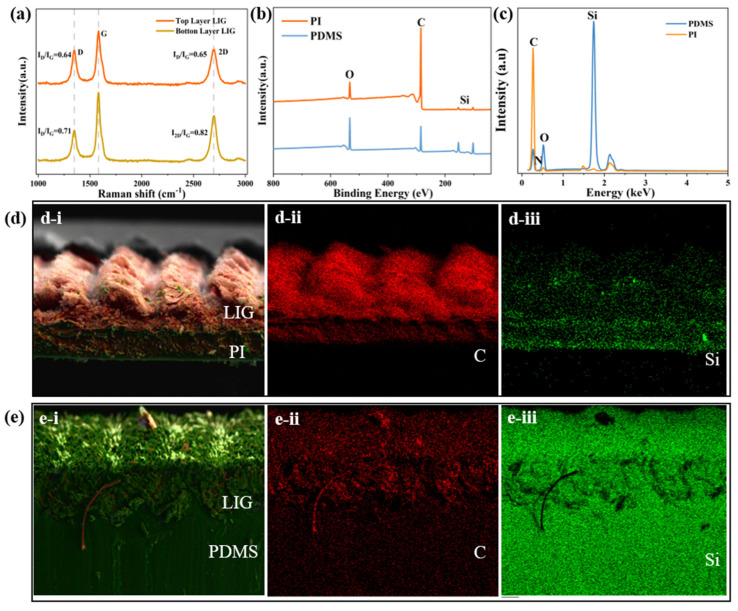
Characterization of the LIG/PDMS-based sensors: (**a**) Raman spectra; (**b**) XPS pattens and atomic content ratios of C, O and Si; (**c**) EDS results including PI and PDMS; (**d**,**e**) SEM-EDS mappings of PI and PDMS: (**d**-**i**), (**e**-**i**) overall mappings; (**d**-**ii**), (**e**-**ii**) carbon element mappings; (**d**-**iii**), (**e**-**iii**) silicon element mappings.

According to the Raman spectral results presented in Figure 3a, graphene corresponds to three characteristic peaks. Specifically, the D peak is observed at approximately 1348 cm^−1^, the G peak appears near 1583 cm^−1^, and the 2D peak is located at 2700 cm^−1^. The presence of these bands reflects the sp^2^ hybridized planar structure of graphene carbon atoms. The intensity of the G peak can be utilized to determine the thickness of the graphene layer. The D peak serves as an indicator of structural defects within the graphene lattice, while the 2D peak confirms the materials’ identity. Notably, the ratio of I_D_/I_G_ is inversely proportional to the degree of crystallization of graphene, that is, a higher I_D_/I_G_ value indicates a greater number of defects and a lower degree of graphitization. Conversely, the I_2D_/I_G_ ratio correlates positively with graphene quality: a higher value indicates superior quality, whereas a lower value suggests the presence of more graphene layers. Raman spectroscopy was performed on graphene samples from different locations, revealing that the overall properties of the graphene were consistent. However, the top layer of graphene exhibited greater thickness, fewer defects, and slightly better quality, whereas the bottom layer demonstrated more defects but thinner layering.

At the same time, we conducted an analysis of the elemental composition of the cross-section before and after the graphene transfer, as shown in Figure 3b–e. PI does not contain silicon (Si), whereas PDMS does. The X-ray photoelectron spectroscopy (XPS) spectra before and after the LIG transfer are shown in Figure 3b. Prior to the transfer, the carbon atoms in the graphene were predominantly replaced by silicon (Si) atoms post-transfer. Given that surface elements are detected during the scanning processing, the Si content appears relatively elevated. As shown in Figure 3c, the carbon (C) content was 90.96% before transfer, decreasing to 57.38% after transfer, yet it still constitutes a relatively high proportion. The sensing portion of the sensor ensures its sensitivity. PI exhibits a relatively high carbon content. Following transfer to PDMS, the contents of oxygen and silicon increased due to the inherent composition of PDMS, resulting in a corresponding decrease in carbon content. The energy-dispersive X-ray spectroscopy (EDS) cross-sectional scans before and after the graphene transfer are shown in Figure 3d,e. Due to the porous structure of graphene, liquid PDMS adhered closely to it and penetrated into graphene upon solidification, covering the surface region of the graphene. Consequently, the bottom layer and LIG before transfer were primary composed of the C element, but post-transfer, the surface and interior were basically covered by PDMS, with Si being the most abundant element, as shown in Figure 3e.

## 3. Results and Discussion

### 3.1. Performance Testing of Flexible Sensors

In the performance test section, the resistance of the prepared strain sensor was evaluated both before and after printing. Prior to transfer, the resistance was approximately 26 Ω, whereas it increased to 48 Ω post transfer. Upon further analysis, the resistivity of graphene was found to be in the order of 10^−6^ Ω·cm, while that of PDMS was 2.914 Ω·cm. The porous graphene, which retained its original three-dimensional structure, contained a significant number of voids. These voids facilitated the formation of conductive pathways when slightly displaced. However, upon infiltration by PDMS, some of these voids became filled, leading to an increase in sensor resistance due to the relatively high resistivity of PDMS. The primary component of the sensor is a small square measuring 10 mm × 10 mm. Following tensile testing, the maximum deformation achieved was 4 mm, corresponding to a strain of 40%.

**Figure 4:**
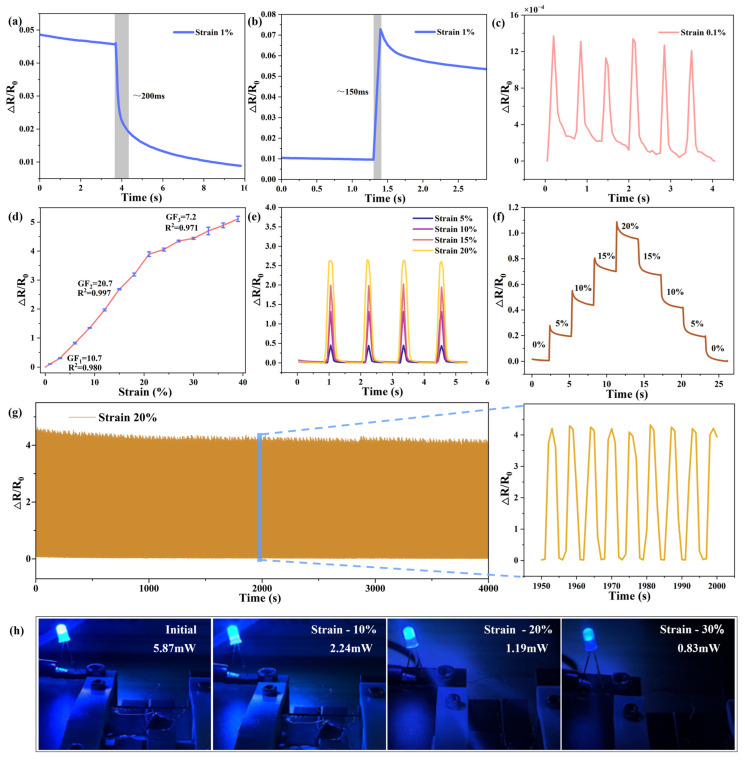
Performance of the flexible wearable strain LIG/PDMS−based sensor. (**a**) Response time of the sensor during releasing. (**b**) Response of the sensor during stretching. (**c**) Minimum detection limit test. (**d**) Sensor sensitivity test. (**e**) Comparison of waveforms at different strains. (**f**) Sensor response under stretching and contraction. (**g**) Cyclic repeat test. (**h**) Brightness changes in LED with different tensile degrees as current-limiting resistance.

However, due to the inferior strain capacity of graphene compared to PDMS, a significant number of cracks and gaps appear in the graphene structure once it exceeds a specific tensile threshold. This results in reduced sensitivity. The most important parameter for flexible wearable strain sensors is their sensitivity, which quantifies a sensor’s responsiveness to external forces and deformations, thereby reflecting its detection capability. The relationship between the gauge factor (GF) of the sensor, its resistance value, and the applied strain can be obtained from the following equation:

(1) $$\;GF=\frac{\left(R-R_{0}\right)/R_{0}}{\varepsilon }$$

where *GF* denotes the sensitivity of the sensor, *R* represents the current resistance of the sensor, and *R*_0_ refers to the resistance of the initial testing state of the sensor, that is, the resistance value under an unloaded condition. The term (*R*−*R*_0_)/*R*_0_ reflects the relative resistance change in the sensor, while ε represents the tensile strain of the sensor. Subsequently, various performance tests and evaluations were performed on the sensors, and the results are shown in Figure 4.

The performance of the LIG/PDMS-based strain sensor was systematically investigated. First, the sensor was securely fixed on a motion testing apparatus. The sensor exhibited consistent resistance variations under identical tensile conditions, indicating its capacity to sustain excellent elastic deformation and robustness. In the performance evaluation of the LIG strain sensor, the relaxation and response time characteristics were investigated, as shown in Figure 4a and Figure 4b, respectively. The sensor exhibited a relaxation time of approximately 200 ms (Figure 4a) during release and a response time of around 150 ms (Figure 4b) during stretching. Furthermore, it is demonstrated in Figure 4c that the sensor achieved a detection limit as low as 0.1%. Notably, it retained the capability to detect subtle surface deformations even after six consecutive stretching cycles, reducing the probability of random errors. As further demonstrated in Figure 4d, the sensor stage-dependent sensitivity. Specifically, the gauge factor (GF) was 10.7 within the range from 0% to 1% strain range and increased to 20.7 between 1% and 21%. When the strain exceeds 21%, the bottom graphene conductive network becomes susceptible to fracture, leading to the failure of the majority of conductive pathways. Consequently, the conductive performance primarily depends on the few remaining connected paths within the upper layer structure, thereby resulting in a significant decrease in the gauge factor (GF). To ensure consistency between the waveform and linearity of the sensor across different tensile degrees, comparisons were made for the stretched waveform at 5%, 10%, 15%, and 20% tensile degrees, as shown in Figure 4e. The results indicate that the waveform under varying tensile conditions is clear and consistent in shape. Gradual stretching and withdrawing tests were also carried out, as shown in Figure 4f, and the resistance value of the sensor remained consistent upon release after stretching, given the same deformation. The gradually decreasing value in the figure can be attributed to the rubber-like characteristics of PDMS, which exhibits a rebound characteristic post-stretching, thereby causing the sensor’s deformation to not remain fixed.

We further performed repeatability tests on the sensor, subjecting it to repeated stretching over a 4000 s, duration as shown in Figure 4g, with a cumulative total of more than 1000 stretching cycles. Upon completion of the test, the sensor maintained its repeatability, reflecting its stability and suitability for high-intensity, repetitive tensile testing scenarios. Additionally, as shown in Figure 4h, the sensor was demonstrated as a current-limiting resistor in a series circuit with a small LED. The brightness of the LED varies under different levels of tensile strain, indicating the sensor’s capacity to modulate resistance in response to deformation. This highlights the potential of the flexible strain sensor for diverse practical applications, attributed to its excellent electromechanical performance. A comparative analysis with other flexible strain sensors is presented [35,36,37,38,39,40,41,42,43] in Table 1. The sensor exhibits exceptional sensitivity and can endure tensile strains of 40%, significantly surpassing the maximum strain of less than 3% for PI and far exceeding the 13% strain typically required for human skin. Notably, the sensor achieves the highest sensitivity within the 1–21% range, reaching a value of 20.7. Sensitivity and tensile capability are primarily constrained by the properties of LIG; thus, increasing the thickness of LIG could improve both the sensitivity and the maximum linear range of this sensor.

### 3.2. Application of LIG/PDMS-Based Flexible Wearable Sensors

Flexible wearable sensors exhibit a broad spectrum of application domains, and their high sensitivity renders them promising for detecting subtle signals on the surface of the body. As part of the latest advancements in non-invasive health monitoring, these sensors can track various physiological parameters and body movement frequencies in real time. Depending on the application scenarios, the resistance variation trends of the sensor differ, as does the corresponding output waveform. To investigate whether the output and responsiveness of a flexible wearable sensor can meet demands under varying tensile stimuli, this study conducted relevant tests on the flexible wearable sensor applied to different parts of a civil aviation test pilot’s body.

**Figure 5:**
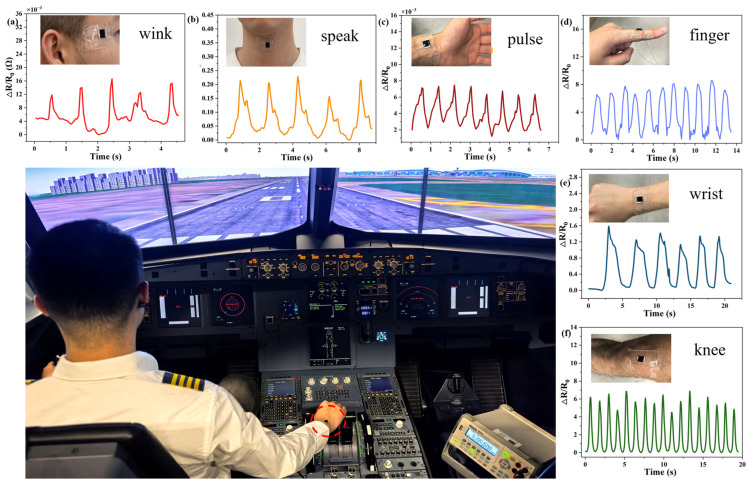
The LIG/PDMS-based sensor is applied to detect various human health signals: (**a**) the blink waveform employed for testing the corner of the eye; (**b**) changes in sensor resistance during speaking; (**c**) pulse detection signals; (**d**) resistance variations when the finger is bent; (**e**) sensor resistance changes upon wrist movement; (**f**) alteration in the sensor’s resistance value when the knee is bent.

PDMS exhibits outstanding biocompatibility with the human body, and no adverse reactions were observed during the initial several hours of sensor attachment to the skin. Consequently, as shown in Figure 5, we investigated the application of the sensor in a test pilot operational scenario. The acquired waveforms indicate that the sensor is highly effective in detecting subtle physiological micro-movements, indicating its potential as an efficient and convenient tool for the real-time monitoring of test pilots. Notably, the detection component of the sensor consists of a compact 10 mm × 10 mm graphene piece positioned centrally, which ensures minimal weight and prevents the imposition of any burden during the detection of fine physiological changes. For example, test pilots may experience fatigue during operation. To a certain extent, the risk of fatigue-related incidents can be mitigated by monitoring whether the blink rate exceeds the normal threshold, as illustrated in Figure 5a. Simultaneously, the sensor depicted in Figure 5b is capable of detecting vocal cord vibrations, thereby facilitating the communication of simple commands within the cockpit. As demonstrated in Figure 5c, sensors can be attached to the test pilot’s hand for continuous monitoring of pulse signals. Furthermore, sensors were strategically applied to various joint motion components operated by test pilots, as shown in Figure 5d–f. The output amplitudes of sensors, corresponding to distinct operating angles and bending degrees, enable the collection of operational amplitudes data. These data can be combined with video data storage to obtain and analyze extensive datasets, ultimately optimizing the driving actions of test pilots. In conclusion, sensors can be effectively applied to the real-time monitoring of test pilots.

## 4. Conclusions

In this study, we propose the development of a LIG/PDMS-based flexible strain sensor for the health monitoring of test pilots. The sensing component of the sensor employs graphene with a three-dimensional porous structure, while the precursor material is composed of PDMS stretchable material. The graphene can be directly generated on PI substrates using laser direct writing technology, allowing for customizable patterns based on practical requirements. This method is environmentally friendly, cost-effective, and straightforward. The prepared graphene exhibits a striped morphology. Upon transferring the graphene onto PDMS, some electrical conductivity is sacrificed to enhance its stretchability. Performance evaluations reveal that the sensor demonstrates excellent robustness, fast response, a low detection limit, a gauge factor of 20.7, and a maximum tensile strain of 40%. Therefore, the developed flexible strain sensor is suitable for the real-time monitoring of physiological signals such as pulse, hand movements, and eye blinks of the test pilot.

## Figures and Tables

**Table 1:** Comparative analysis of performance for flexible strain sensors with various materials.

Materials	Processing	Response Time (ms)	Gauge Factor (GF)	References
LIG-PDMS	Laser direct writing	150	15.79	[35]
PAM-3-TSASN-LiCl hydrogel	Surface modification	210	4.5	[36]
GAF	Graphite oxide reduction	208	0.84	[37]
LM@CNTs-5/Fe/Ecoflex	4D Printing	83	19.8	[38]
B-TMN-SHFSS	\	230	0.146	[39]
LIG	Laser direct writing	150	41.4	[40]
PDMS-SEBS(CB)	Spraying carbon black	168	7.35	[41]
Gallium-based liquid metals	Extrusion thermal pressing method	180	4.59	[42]
PDMS/CNT/FM@MX	Ultrasonically dispersed	330	5.59	[43]
LIG-PDMS	Laser direct writing	150	20.7	This work

## Data Availability

Data are contained within the article.
